# Discussion of Dr. Jones’ Paper

**Published:** 1907-07

**Authors:** 


### DISCUSSION ON DR. JONES' PAPER.

Dr. J. N. Ellis said that the essayist had presented the subject so thoroughly that he had little to add. The question is a very interesting one, as it is claimed that there are 2,000,000 patients with gall stones at all times in Germany alone. Many cases are not recognized as such, but are treated for other diseases. The etiology, as Dr. Jones brought out, is largely dependent upon stasis and infection. He also agreed with the essayist as to the treatment. When diagnosed, the condition is always a surgical one. Medicine may allay catarrhal symptoms, but when stones are present they will always return. The gall stone can rarely be made to pass out. They are frequently located in the ducts. The operation depends upon the individual case. Cholcystotomy appears to relieve many of the patients. Cholcystectomy is not much more difficult and gives excellent results. The mortality with gall stones is low when the operation is done early. It is the delayed operations that carry up the mortality statistics.

Dr. Cyrus Strickler was very much interested in the paper, and thought this disease always surgical. In his experience he had found it a serious condition, as he has seen it associated with malignant disease on several occasions. It may also cause a pancreatitis by blocking the duct. The method of opening and draining seemed to him the operation of choice. If the gall bladder is suspended to afford free flow of bile the stones will not reform.

Dr. E. Bates Block believed that the medical side should be heard from, as he did not think an operation was invariably called for. Slight attacks may not jeopardize the patients life, and may never recur again. Where the symptoms are more pronounced, persistent or of frequent occurrence, no one should hesitate in advising an operation. Where traveling down ducts causing severe symptoms they should be operated upon. Dr. Block does not believc gall stones ever cause carcinoma, and he has never seen a primary carcinoma of the liver. Cholecystitis may give symtoms of gall stones without their being present. Where the bile is thick and inspisated it may plug the ducts as a stone, and mistakes have been made in giving drugs to liquify the bile before raising the blood pressure above that of the bile as should be done to prevent jaundice becoming more marked after the treatment. If the blood pressure is first raised and the bile then liquified by proper medication the jaundice diminishes. Dr. Block is the first to call attention to this point and he thinks it an important one. Large doses of olive oil seem to assist the passage off of gall stones. Carlsbad salts and local counter-irritation are also beneficial. A patient was referred to who had been so treated, who was free from jaundice until just before an operation, and the gall bladder was found to be much distended with stones, and one was found in the common duct which had caused the jaundice just before the operation.

Dr. Jones (closing) showed several specimens exemplifying the different types of gall stones. He realized that some had to be treated with medicine, but urged that the operation be done as early as possible, for it became much more difficult and dangerous after the stone passed into the ducts. Dr. Jones asked Dr. Block what his idea was as to the manner in which olive oil assisted in the passage of gall stones. In case of recurrence of stones he thought this meant they were in the hepatic ducts when the operation was done.

